# ASB3 expression aggravates inflammatory bowel disease by targeting TRAF6 protein stability and affecting the intestinal microbiota

**DOI:** 10.1128/mbio.02043-24

**Published:** 2024-08-20

**Authors:** Mingyang Cheng, Bin Xu, Yu Sun, Junhong Wang, Yiyuan Lu, Chunwei Shi, Tianxu Pan, Wenhui Zhao, Xiaoxu Li, Xiaomei Song, Jianzhong Wang, Nan Wang, Wentao Yang, Yanlong Jiang, Haibin Huang, Guilian Yang, Yan Zeng, Dongqin Yang, Chunfeng Wang, Xin Cao

**Affiliations:** 1 College of Veterinary Medicine, Jilin Agricultural University, Changchun, China; 2 Jilin Provincial Engineering Research Center of Animal Probiotics, Jilin Agricultural University, Changchun, China; 3 Jilin Provincial Key Laboratory of Animal Microecology and Healthy Breeding, Jilin Agricultural University, Changchun, China; 4 Engineering Research Center of Microecological Vaccines (Drugs) for Major Animal Diseases, Ministry of Education, Jilin Agricultural University, Changchun, China; 5 Department of General Surgery, Shanghai 10th People's Hospital, Tongji University School of Medicine, Shanghai, China; 6 Department of Gastroenterology, Chongqing General Hospital, Chongqing, China; 7 Central Laboratory, Huashan Hospital, Fudan University, Shanghai, China; Yale University School of Medicine, New Haven, Connecticut, USA; University of Pennsylvania, Philadelphia, Pennsylvania, USA

**Keywords:** inflammatory bowel disease, ASB3, intestinal epithelial cells, intestinal microbiota, TRAF6

## Abstract

E3 ubiquitin ligase (E3) plays a vital role in regulating inflammatory responses by mediating ubiquitination. Previous studies have shown that ankyrin repeat and SOCS box-containing protein 3 (ASB3) is involved in immunomodulatory functions associated with cancer. However, the impact of ASB3 on the dynamic interplay of microbiota and inflammatory responses in inflammatory bowel disease (IBD) is unclear. Here, we systematically identify the E3 ligase ASB3 as a facilitative regulator in the development and progression of IBD. We observed that ASB3 exhibited significant upregulation in the lesions of patients with IBD. ASB3^−/−^ mice are resistant to dextran sodium sulfate-induced colitis. IκBα phosphorylation levels and production of proinflammatory factors IL-1β, IL-6, and TNF-α were reduced in the colonic tissues of ASB3^−/−^ mice compared to WT mice. This colitis-resistant phenotype was suppressed after coprophagic microbial transfer and reversed after combined antibiotics removed the gut commensal microbiome. Mechanistically, ASB3 specifically catalyzes K48-linked polyubiquitination of TRAF6 in intestinal epithelial cells. In contrast, in ASB3-deficient organoids, the integrity of the TRAF6 protein is shielded, consequently decelerating the onset of intestinal inflammation. ASB3 is associated with dysregulation of the colitis microbiota and promotes proinflammatory factors’ production by disrupting TRAF6 stability. Strategies to limit the protein level of ASB3 in intestinal epithelial cells may help in the treatment of colitis.

**IMPORTANCE:**

Ubiquitination is a key process that controls protein stability. We determined the ubiquitination of TRAF6 by ASB3 in intestinal epithelial cells during colonic inflammation. Inflammatory bowel disease patients exhibit upregulated ASB3 expression at focal sites, supporting the involvement of degradation of TRAF6, which promotes TLR-Myd88/TRIF-independent NF-κB aberrant activation and intestinal microbiota imbalance. Sustained inflammatory signaling in intestinal epithelial cells and dysregulated protective probiotic immune responses mediated by ASB3 collectively contribute to the exacerbation of inflammatory bowel disease. These findings provide insights into the pathogenesis of inflammatory bowel disease and suggest a novel mechanism by which ASB3 increases the risk of colitis. Our results suggest that future inhibition of ASB3 in intestinal epithelial cells may be a novel clinical strategy.

## INTRODUCTION

Inflammatory bowel disease (IBD) is a heterogeneous group of chronic inflammatory diseases, including Crohn’s disease (CD) and ulcerative colitis (UC), which is usually triggered by multiple complex factors (1). During IBD, various immune cell populations in the intestinal mucosa promote an inflammatory response by secreting proinflammatory factors. Meanwhile, intestinal epithelial cells (IECs) play an essential role in regulating intestinal immune homeostasis by maintaining the integrity of the intestinal mucosal barrier and regulating the functions of intestinal immune cells (2). IBD-associated genetic defects, epithelial barrier defects, dysregulated immune response, diet, and antibiotic use, among other factors, lead to early dysbiosis of the gut lamina propria, which may precede the development of clinically overt disease (3). Continued inflammation may lead to late dysbiosis, manifested by an overall reduction in microbial diversity and loss of beneficial symbionts, leading to further exacerbation of the inflammatory response (4). Currently, the treatment of IBD is limited to immunotherapy. Therefore, understanding the host gene-microbiota causality and interactions in colitis provides new insights for developing therapeutic regimens with synergistic microbiota (5).

Previous studies have shown that the E3 ubiquitin ligase, one of the critical regulators of autophagy and apoptosis, plays an essential role in normal development, tumor progression, and tissue homeostasis (6). E3 ubiquitin ligases promote protein degradation through the ubiquitin-proteasome system. One class of E3 ubiquitin ligases is called SOCS family proteins because of the SOCS box domain (7). Ankyrin (ANK) repeat and SOCS box containing protein 3 (ASB3) is a member of the ASB family. This family has 18 members, and the protein structure includes the N-terminal ankyrin repeat and the C-terminal SOCS cassette (8). The N-terminal domain of the ASB3 protein is responsible for substrate recognition, and the C-terminal SOCS box performs E3 enzyme function.

Ubiquitination modification is a crucial posttranslational modification in the inflammatory response, affecting the function of essential proteins of the signaling pathway (9). Dysregulation of E3 ubiquitin ligase-related response processes has been reported in IBD and numerous immune-related diseases (10). For instance, ASB1 promotes TAB2 stability by inhibiting K48-linked polyubiquitination, which leads to enhanced NF-κB and MAPK activation (11). MARCH3 negatively regulates IL-6/STAT3 signaling to limit colitis by targeting IL-6Rα ubiquitination of IL-6Rα at K401 and subsequent degradation (12). In addition, TRIM34 regulates intestinal inflammation by controlling Muc2 cytokinesis in colonic goblet cells (13). Thus, more elucidation of the functions and targets of E3 ubiquitin ligases will provide additional avenues for the treatment of infectious diseases. To date, despite new evidence of the importance of ASB3 regulation in cancer development, its role in colonic inflammation has not been evaluated.

Here, we investigated the physiological role of ASB3 in the intestine and found that ASB3-deficient mice exhibited reduced susceptibility to dextran sodium sulfate (DSS)-induced colitis. Further experiments showed that ASB3 deficiency directly protects Bacteroidota and Verrucomicrobiota and attenuates the overgrowth of Proteobacteria, thus favoring the growth of *Akkermansia* and *Bacteroides*. Moreover, we demonstrate that IECs ASB3 modulates TLR-Myd88/TRIF-independent NF-κB activation by specifically enhancing K48-linked polyubiquitination of TRAF6. This novel mechanism precedes the dysregulation of gut ecology and reinforces the role of ASB3 in maintaining inflammatory signaling and a healthy gut microbial ecology.

## MATERIALS AND METHODS

### Human samples

Human colon samples from IBD patients were provided by the tissue bank in accordance with its regulations and approved by the Ethics Committee of Tongji University and Shanghai Tenth People’s Hospital.

### Mice

ASB3^−/−^ mice were generated using PiggyBac transposon-based targeting technology and were kindly provided by Professor Dongqin Yang (Shanghai Fudan University). Mice used in this study were housed under specific-pathogen-free conditions and had an FVB genetic background. To avoid the effect of familial heritability on the colony, ASB3^−/−^ mice and the wild-type (WT) controls were all generated from the same heterozygous (HE) ASB3^+/-^ parents. WT, HE, and ASB3^−/−^ mice were identified by PCR amplification and DNA sequencing with the primers 5′-CTGAGATGTCCTAAATGCACAGCG-3′, 5′-CCATGACCAAACCCAATTTACACAC-3′, and 5′-TTGTTTTTTTTCCCCCCTAGACAGG-3′, respectively. For the cohousing experiment, 4-week-old mice from the same mother were divided into either individually reared (SiHo) or cohoused with age- and sex-matched mice (CoHo) for 6 weeks. For antibiotic treatment, mice were given an ABX cocktail (33.2 mg ampicillin, 33.2 mg neomycin, 33.2 mg metronidazole, and 16.7 mg vancomycin) orally for five consecutive days (14).

### Induction of DSS-mediated acute colitis

Six- to eight-week-old sex-matched mice were induced to develop acute colitis by 3.0% (wt/vol) DSS (MW 36–50 kDa; MP Biomedical) dissolved in sterile, distilled water *ad lib* for the experimental days 1–6 and were then provided regular water for another 2 days (15). The DSS solution was made freshly every day. The severity of colitis was assessed by the Disease Activity Index (DAI), including daily weight loss, stool consistency, and occult blood, as previously described (16). Briefly, weight score is as follows: 0 means no weight loss, 1 means 1%–5% weight loss, 2 means 5%–10% weight loss, 3 means 10%–20% weight loss, and 4 means more than 20% weight loss. The consistency of the stool is scored as follows: 0 indicates normal stool pattern, 1 indicates semi-formed stool not adhering to the anus, 2 indicates semi-formed stool adhering to the anus, and 3 indicates liquid stool adhering to the anus. Bleeding was scored as follows: 0 indicates blood occult negative, 1 indicates blood occult positive, 2 indicates blood traces in the stool, 3 indicates obvious blood traces in the anus, and 4 indicates rectal hemorrhage. Weight score, stool consistency score, and bleeding score were added and expressed as clinical scores. The entire colon was removed on day 8 to measure the colon length.

### Cells, organoids, and plasmids

Human embryonic kidney cells (HEK293T) and human colorectal adenocarcinoma cells (HT-29) were cultured in Dulbecco’s modified Eagle’s medium (DMEM) and RPMI-1640 supplemented with 10% (vol/vol) fetal bovine serum (FBS) and 2 mM glutamine with penicillin (100 U/mL)/streptomycin (100 mg/mL). Colon intestinal epithelial cells or crypts were dissociated from colonic segments of WT or ASB3^−/−^ mice. Isolated colonic crypts were resuspended in DMEM/F12 medium, counted, and resuspended in colonic organoid growth medium and matrix gel (Corning) at a 1:1 ratio (17). The medium was changed every other day, and after 7 days, the organoid was stimulated with 150 ng/mL mouse recombinant TNF-α (PeproTech) for 24 h (18). All cells were cultured and maintained at 37°C with 5% CO_2_.

Plasmids for Flag-tagged Myd88, TRIF, TRAF3, TRAF6, TAB1, IKKβ, Myc-tagged ubiquitin (Myc-K48), NF-κB-Luc, and pRL-TK (internal control luciferase reporter plasmid) used in the study were described previously (19). Plasmids for HA-tagged ASB3, ASB3 (ΔANK), and ASB3 (ΔSOCX) were kindly provided by Prof. Dongqin Yang (Fudan University, Shanghai, China). Plasmids for Flag-tagged TRAF6 (aa 260–522), TRAF6 (aa 110–522), and TRAF6 (aa 1–349) were kindly provided by Prof. Qiyun Zhu (Lanzhou Veterinary Research Institute, Chinese Academy of Agricultural Sciences, China).

### Histology

For histopathological analysis, colon tissue from WT and ASB3^−/−^ mice was fixed in 4% paraformaldehyde solution overnight for processing and embedded in paraffin wax according to standard procedures. Then, 3 µm sections were taken and stained with hematoxylin and eosin or Alcian blue. The stained sections were scanned with a microscope (Leica).

### Confocal microscopy

Confocal microscopy was performed as previously described (20). The indicated plasmids were cotransfected into 293T cells and collected after 24 h. Cells were fixed with 4% paraformaldehyde for 15 min at room temperature and subsequently blocked and permeabilized with 0.5% Triton X-100 5% skim milk for 1 h at 4°C. After blocking, slides for immunofluorescence were incubated with fluorescein-conjugated secondary antibodies (1:500) overnight at 4°C in the dark and mounted with DAPI (Beyotime) (1:1,000). Images were acquired with a Zeiss microscope (LSM 710) to visualize stained cells.

### Antibodies and reagents

The antibodies used in this study were as follows: HRP-conjugated anti-HA (12013819001) and Myc (11814150001) antibodies (Roche); phosphorylated IκBα (AF5851), IκBα (AG2737), HRP-labeled goat anti-mouse IgG (H+L) (A0216), and HRP-labeled goat anti-rabbit IgG (H+L) (A0208) antibodies (Beyotime); FITC goat anti-mouse IgG (H+L) (K1201) and Cy5 goat anti-rabbit IgG (H+L) (K1212) secondary antibodies (APExBIO); anti-TRAF3 (66310-1-Ig), anti-HA (66006-2-Ig), anti-DYKDDDDK (20543-1-AP), anti-β-actin (66009-1-Ig), anti-NF-κB p65 (66535-1-Ig), and anti-villin (16488-1-AP) antibodies (Proteintech); anti-TRAF6 antibody (8028), anti-ubiquitin (K48) (8081), and HRP-conjugated mouse anti-rabbit IgG (Conformation Specific) (5127) antibodies (Cell Signaling Technology); anti-ubiquitin (WT) antibody (Santa Cruz, sc-8017); anti-ASB3 (88812) antibody (MBL); anti-TRAF6 antibody (ab137452) and anti-EpCAM antibody (ab71916) (Abcam); HRP-conjugated anti-Flag (A8592) antibody (Sigma). Reagents used in the study included 3-methyladenine (M9281), MG132 (M7449), DMSO (D2650), NH_4_Cl (09718), anti-Myc agarose affinity beads (A7470), and protein A/G agarose affinity beads (P6486/E3403) (Sigma); mouse IL-1β/IL-6/TNF-α ELISA kit (MM-0040M1/MM-0163M1/MM-0132M1) (Meimian); human and mouse recombinant TNF-α (300-01A) (315-01A) (PeproTech); DAPI (C1005), ZVAD (caspase inhibitor Z-VAD-FMK, C1202), NP-40 (ST366), and HEPES Solution (C0215) (Beyotime). TnT T7/SP6 Quick Coupled Transcription/Translation System (L1170/L2080) (Promega).

### Coimmunoprecipitation

HEK293T cells at 80%–90% confluency were washed with PBS three times and collected using a cell scraper into tubes. The same mass of colonic tissue was washed three times with precooled PBS and mechanically homogenized with RIPA lysis buffer (Thermo Scientific) containing 1% phenylmethylsulfonyl fluoride (PMSF). All samples were lysed in NP-40 lysis buffer containing 20 mM Tris-HCl (pH 8.0), 1 mM EDTA, 1% NP-40, and 150 mM NaCl supplemented with Halt Protease Inhibitor Cocktail and kept on ice for 30 min. After 30 min of incubation, the lysates were spun down at 12,000 rpm at 4°C for 20 min. All supernatants were collected, and protein was quantified using the BCA Protein Assay Kit (Beyotime). The supernatants were pretreated with 30 µL of anti-Flag agarose affinity gels or protein A/G at 4°C for 2 h. The indicated primary antibody or IgG control (mouse IgG, Beyotime) was then added to the pretreated lysates and incubated overnight at 4°C. The IP complexes and whole-cell lysates were subjected to protein transfer by the conventional Western blotting method, and protein development was detected using the above-mentioned antibodies.

### Colon IECs and LPLs isolation and flow cytometry analysis

The colon IECs and lamina propria lymphocytes (LPLs) were isolated as described previously (21–23). Briefly, the colon was opened longitudinally in pre-chilled PBS to expose the lumen of the tube. Colon tissue was gently rinsed and the intestinal contents were removed and cut into small 1 cm pieces. Tissue pieces were incubated in a pre-digested medium (RPMI-1640 containing 20 mM HEPES, 5 mM EDTA, 1% penicillin and streptomycin, 2% FBS, and 2 mM DTT) for 20 min at 37°C and 180 rpm in a shaking incubator. The contents were shaken vigorously for 2 min and washed with PBS. The epithelial cells in the supernatant were filtered with a 40 µm strainer and spun down. IECs were collected from the interphase. The remaining tissues were transferred to digestion buffer (RPMI-1640 supplemented with 1% penicillin and streptomycin, 20 mM HEPES, 1.5 mg/mL collagenase IV, 0.5 mg/mL DNase I, and 2% FBS) and incubated in a shaking incubator at 37°C, 250 rpm for two consecutive 15 min. Cells were washed and collected through a 70 µm cell membrane. After filtering, leukocytes were purified on Percoll density-gradient separation. Purified cLPs were washed and stimulated with 50 ng/mL phorbol 12-myristate 13-acetate (PMA) (Sigma) and 500 ng/mL ionomycin (Sigma) in the presence of GolgiPlug (BD) for 4 h prior to staining for flow cytometry analysis by LSR Fortessa (BD). Fluorochrome-conjugated antibodies used in this study were as follows: Fixable Viability Stain 780 (565388), biotin-labeled TCR β Chain (553168), biotin-labeled γδT-cell receptor (553176), biotin-labeled CD11b (557395), biotin-labeled CD19 (553784), biotin-labeled Ly-6G and Ly-6C (553124), biotin-labeled TER-119 (553672), streptavidin APC-Cy 7 (554063), CD16/32 (553140), CD45.1 (550994), CD3 (557984), CD4 (561115), CD8 (553035), CD44 (560567), CD25 (553075), Nkp46 (560756), CCR6 (557976), RORgt (562607) (BD Biosciences); IL-22 (516409) (Biolegend). Cell surface staining was performed by incubating cells with antibodies for 30 min at 4°C after blocking with CD16/32. RORgt and IL-22 staining was carried out using an intracellular transcription factor kit (eBioscience) or cytokine staining kit (BD Biosciences).

### 16S rDNA sequencing

16S rDNA amplicon libraries were produced from the DNA of feces and were completed by Tianjin Novogene Bioinformatics Technology Co. Ltd. (Tianjin, China). Total genome DNA from samples was extracted using the CTAB/SDS method. DNA concentration and purity were monitored on 1% agarose gels. According to the concentration, DNA was diluted to 1 ng/µL using sterile water. PCR amplification of the V3–V4 region of the bacterial 16S rRNA gene was performed using specific primers with Barcode for the 16S V4 region primer 515F-806R based on the selection of the sequenced region. Sequencing libraries were generated using Illumina TruSeq DNA PCR-Free Library Preparation Kit (Illumina, USA) following the manufacturer’s recommendations, and index codes were added. The library quality was assessed on the Qubit 2.0 Fluorometer (Thermo Scientific) and Agilent Bioanalyzer 2100 system. At last, the library was sequenced on an Illumina NovaSeq platform and 250 bp paired-end reads were generated.

### RNA-seq analysis

Colon tissues collected from WT and ASB3^−/−^ mice treated with drug administration were used for RNA-seq analysis. The whole sequencing process was carried out by Tianjin Novogene Bioinformatics Technology Co. Ltd. (Tianjin, China). Total RNA was isolated using the Trizol Reagent (Invitrogen). Total amounts and integrity of RNA were assessed using the RNA Nano 6000 Assay Kit of the Bioanalyzer 2100 system (Agilent Technologies, CA, USA). Total RNA was used as input material for the RNA sample preparations. Briefly, mRNA was purified from total RNA by using poly-T oligo-attached magnetic beads.

### Dual-luciferase reporter assays

Plasmids encoding Flag-tagged MyD88, TRAF6, and IKKβ or HA-tagged ASB3 were co-transfected with NF-κB-Luc and pRL-TK into HEK293T cells for 24 h using Lipofectamine 3000 (Invitrogen) reagent. Cell samples were collected at the indicated times and lysed or assayed using the Dual-Luciferase Reporter Gene (DLR) Assay System (Promega) according to the manufacturer’s instructions. Finally, the relevant reporter gene activity is assayed with Firefly luciferase and *Renilla* luciferase reagents.

### Quantitative real-time RT-PCR

RNA was isolated with Trizol reagent (Takara). cDNA was synthesized using the reverse transcription Moloney mouse leukemia virus reverse transcriptase (Promega). Real-time PCR was performed using SYBR Green Mix (Takara). Reactions were run with the Applied Biosystems 7500 real-time PCR System. The results were displayed as relative expression values normalized to GAPDH. Sequences of PCR primers are as follows: 5′-CTGATAACAGGGGATGGATCC (forward primer for Hu ASB3), 5′-CGAGATGCAAAGCACAGAAAC (reverse primer for Hu ASB3), 5′-ACAGAGGCTTACTCAGACACG (forward primer for mus asb3), 5′-TCCCCTGTTATCAGCGACATC (reverse primer for mus asb3), 5′-GCAACTGTTCCTGAACTCAACT (forward primer for mus IL-1b), 5′-ATCTTTTGGGGTCCGTCAACT (reverse primer for mus IL-1b), 5′-TCTGCAAGAGACTTCCATCCAGTTGC (forward primer for mus IL-6), 5′-AGCCTCCGACTTGTGAAGTGGT (reverse primer for mus IL-6), 5′-TCAGGTGCAAGGTGAAGTTG (forward primer for mus Reg3g), 5′-GGCCACTGTTACCACTGCTT (reverse primer for mus Reg3g), 5′-ATGCCCACCTCCTCAAAGAC (forward primer for mus Muc2), 5′-GTAGTTTCCGTTGGAACAGTGAA (reverse primer for mus Muc2), 5′-AAAATCAAGTGGGGCGATGCT (forward primer for Hu GAPDH), 5′-GGGCAGAGATGATGACCCTTT (reverse primer for Hu GAPDH). 5′-ACGGCCGCATCTTCTTGTGCA (forward primer for mus GAPDH) and 5′-ACGGCCAAATCCGTTCACACC (reverse primer for mus GAPDH).

### Immunoblotting analysis

Colon was dissected longitudinally and washed with cold PBS. Sections of 3 cm distal to each colon or 293T cells were collected and mechanically homogenized with PRIA lysis buffer (Thermo Scientific) containing 1% PMSF according to the manufacturer’s protocol. The supernatant was collected and boiled in an SDS sample buffer for 10 min and analyzed by immunoblotting. Immunoblots were observed using Amey Imager 600RGB and quantified using ImageJ.

### Statistical analysis

Data are presented as the mean ± SD unless otherwise indicated. All samples were analyzed using GraphPad Prism 8 software. Data were analyzed by unpaired two-tailed Student’s *t* test or two-way ANOVA with Sidak’s test to correct for multiple comparisons. Probability (*P*) values of <0.05 were considered significant: *P < 0.05, **P < 0.01, ***P < 0.001, and ****P < 0.0001; n.s., not significant.

## RESULTS

### ASB3 expression is upregulated in IBD patients and mice with DSS-induced colitis

**Fig 1:**
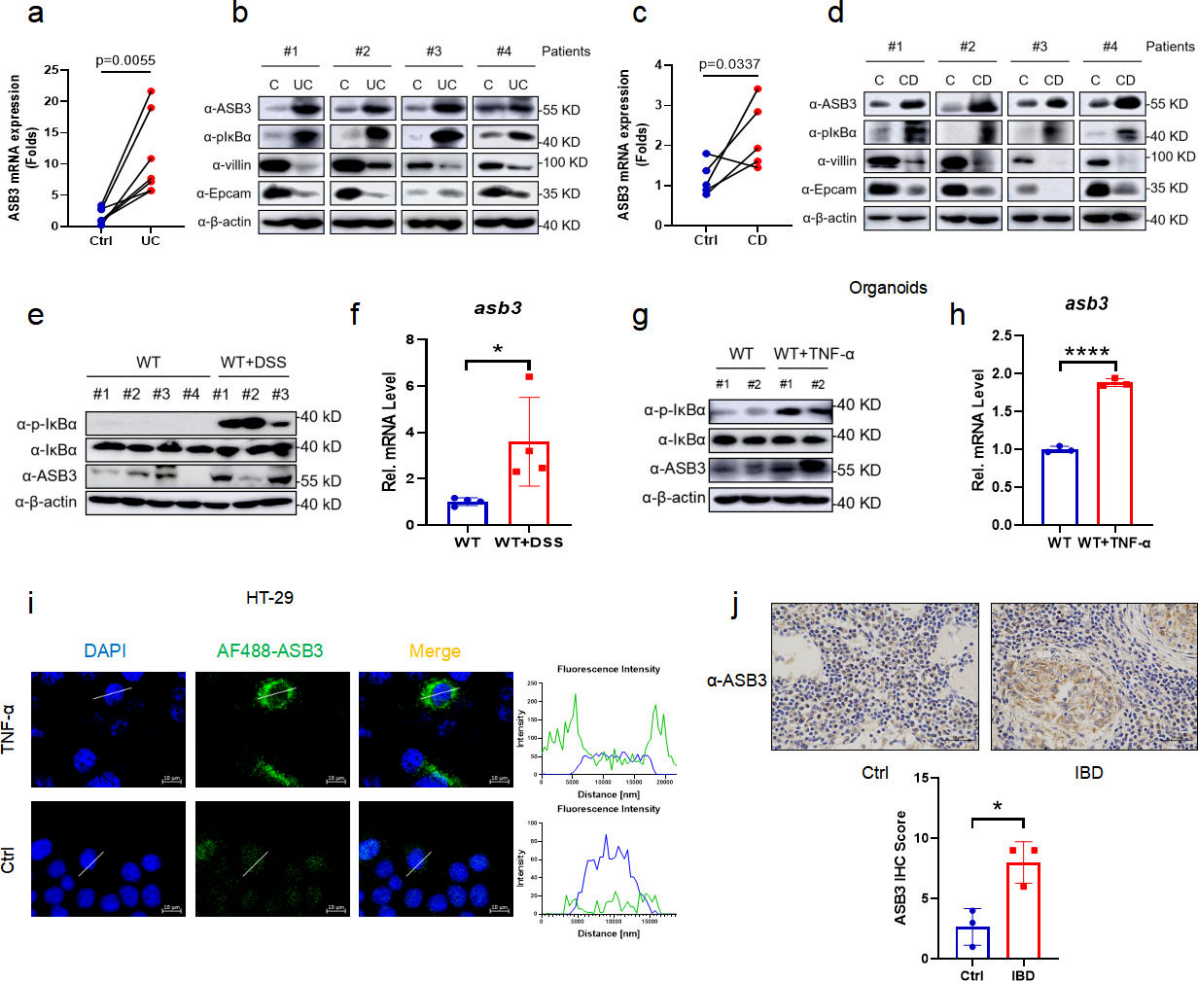
Abnormal ASB3 expression levels in IBD patients and mice with DSS-induced colitis. (a) The mRNA expression of ASB3, IL-1β, IL-6, and TNF-α was measured in both UC groups (*n* = 7) relative to controls. (b) Representative immunoblotting of p-IκBα, IκBα, ASB3, villin, EpCAM, and β-actin protein expression in UC. (c) The mRNA expression of ASB3, IL-1β, IL-6, and TNF-α was measured in both CD groups (*n* = 5) relative to controls. (d) Representative immunoblotting of p-IκBα, IκBα, ASB3, villin, EpCAM, and β-actin protein expression in CD. (e) The expression of p-IκBα, IκBα, ASB3, and β-actin proteins in the colons of DSS-treated or untreated mice was detected by Western blotting. (f) The mRNA expression levels of ASB3 in the colons of DSS-treated or untreated mice were determined by quantitative real-time RT-PCR (qPCR) assay. (g) The expression of p-IκBα, IκBα, ASB3, and β-actin proteins in TNF-α-treated or untreated organoids was detected by Western blotting. (h) The mRNA expression levels of ASB3 in the organoids of TNF-α-treated or untreated mice were determined by qPCR assay. (i) HT-29 cells were treated with TNF-α (150 ng/mL) for 12 h and then stained with indicated antibody and secondary antibody. The nuclei were stained by DAPI. Scale bars, 50 µm. (j) Representative IHC staining and quantification of ASB3 in colon tissues collected from IBD patients. Scale bars, 50 µm. *P* values less than 0.05 were considered statistically significant (*P < 0.05, **P < 0.01, ***P < 0.001, and ****P < 0.0001) in panels a and c by paired Student’s *t* test and in panels c, f, h, and j by unpaired Student’s *t* test.

To explore the potential role of ASB3 in IBD, we examined the expression levels of ASB3 in the colonic tissues of UC and CD patients. All paired samples of Crohn’s disease or ulcerative colitis were obtained from the same patient’s colonic lesion site (UC or CD) and adjacent uninflamed tissue (control). The results showed that the mRNA expression of ASB3 was upregulated in both UC (*n* = 7) and CD (*n* = 5) compared to controls (Fig. 1a and c). Consistent with the transcriptional level, the expression of ASB3 protein was enhanced at the site of IBD inflammation and was accompanied by damage to the intestinal barrier (Fig. 1b and d). We then verified the level of ASB3 in the colons of mice. There was an increase in IκBα phosphorylation and ASB3 expression in the colonic tissue from DSS-treated FVB mice (Fig. 1e and f). Furthermore, similar results were observed in colon organoids and HT-29 cells stimulated with TNF-α (Fig. 1g through i), suggesting that intestinal inflammation is accompanied by abnormally high ASB3 expression. We also observed that ASB3 expression levels were higher in the colonic mucosa of IBD patients than in the colonic mucosa of the uninflamed site (Fig. 1j). In conclusion, abnormally high expression of ASB3 was positively correlated with inflammation in IBD tissues.

### ASB3^−/−^ mice are resistant to DSS-induced colitis

**Fig 2:**
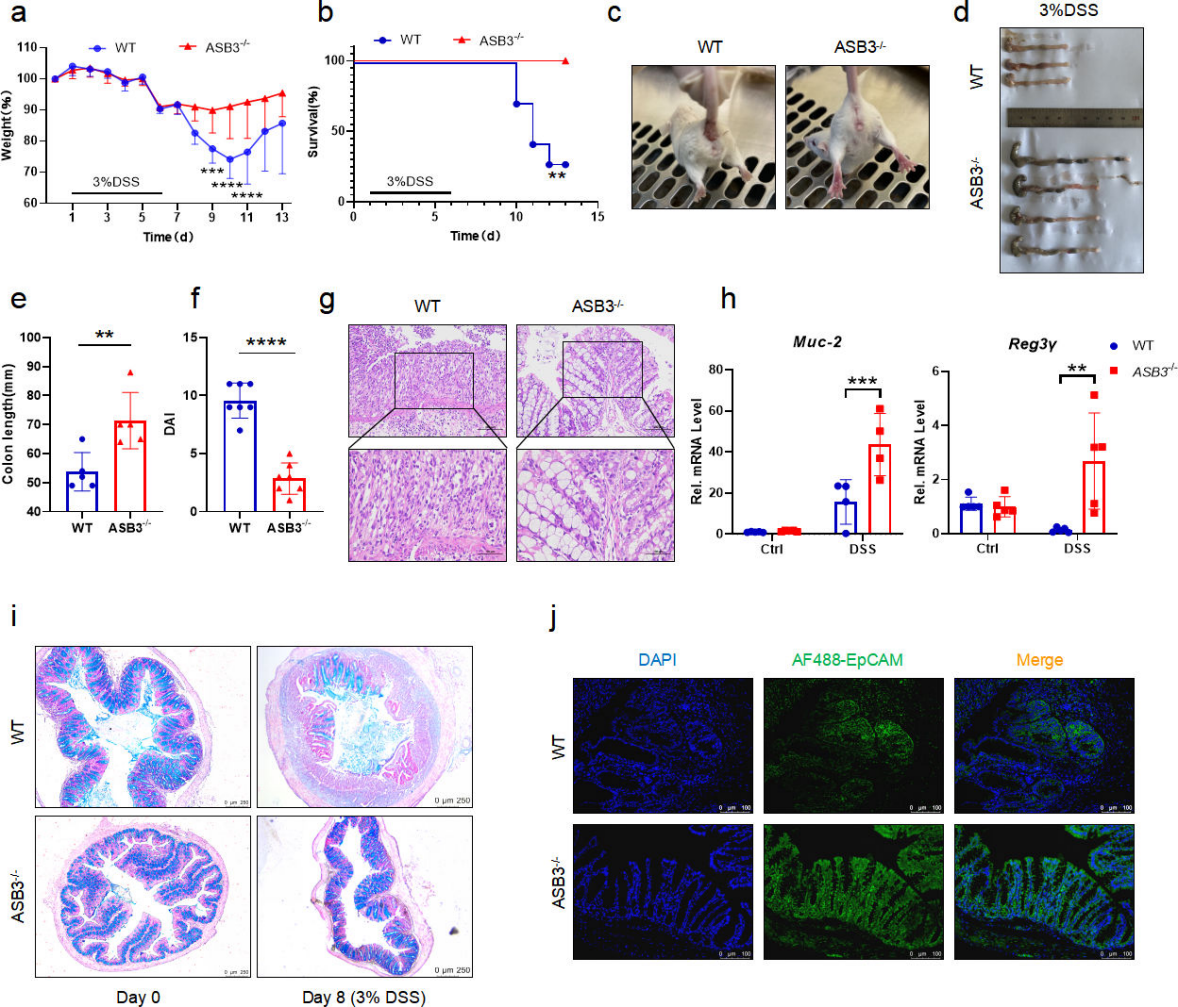
ASB3 deficiency provides protection against DSS-induced colitis. (**a and **b) Body weight (a) and survival (b) of conventionally raised wild-type and ASB3^−/−^ mice treated with 3% DSS (above horizontal axes) (WT, *n* = 7; ASB3^−/−^, *n* = 7); the results are presented relative to initial values, set as 100% (throughout). (c) Representative images of diarrhea/bloody stools observed on day 6. (**d and **e) Colon length on day 8. (f) DAI scores were determined on day 6. (g) Representative images of pathological H&E-stained colon sections collected on day 8. Scale bar, 100 or 50 µm. (h) The mRNA expression levels of Muc-2 and Reg3γ were determined by qPCR assay. (i) Representative images of Alcian Blue staining of colon tissues of WT and ASB3^−/−^ mice. Scale bar, 250 µm. (j) IF for EpCAM in the colon; representative image of three mice/genotype. Scale bar, 100 µm. Differences between groups in panels e and f were determined by unpaired Student’s *t* test, in panels a and h by one-way ANOVA, and in panel b by log-rank test.

To further investigate the function of ASB3 in the development of colitis, ASB3^−/−^ and WT mice were stimulated with 3% DSS for 6 days and then allowed to recover with normal drinking water for 2 days. The lack of ASB3 expression in the healthy state did not lead to anatomical abnormalities or spontaneous inflammation of the intestine (Fig. S1b through d). Nevertheless, our results showed that ASB3^−/−^ mice had a significantly lower rate of weight loss than WT mice after the DSS challenge (Fig. 2a). We observed that WT mice died during the acute phase of DSS challenge, while all ASB3^−/−^ mice survived (Fig. 2b). In addition, the reduced severity of colitis observed in ASB3^−/−^ mice was manifested by less diarrhea and fewer bloody stools, longer colonic length, and reduced clinical scores (Fig. 2c through f). To assess this hypoinflammatory phenotype, we analyzed histopathological sections of the colon. Consistent with the clinical features described above, ASB3^−/−^ mice exhibited less severe submucosal inflammatory cell infiltration and less severe epithelial or crypt erosion than WT mice (Fig. 2g). Epithelial cells include enterocytes, goblet cells, enteroendocrine cells, Paneth cells, and M cells, which are tightly connected and covered with mucus. Intestinal barrier damage and epithelial cell depletion are regarded as important characteristics associated with mucosal inflammation (24). We further confirmed by quantitative real-time RT-PCR (qPCR) and Alcian blue staining that ASB3 expression facilitated a reduction in the number of goblet cells (Fig. 2h and i). We also found that the absence of ASB3 expression during inflammation attenuated the damage to intestinal epithelial cells (Fig. 2j). Our findings suggest that ASB3 deficiency protects against DSS-induced colonic inflammation.

### ASB3 exacerbates colitis by promoting the release of proinflammatory factors

**Fig 3:**
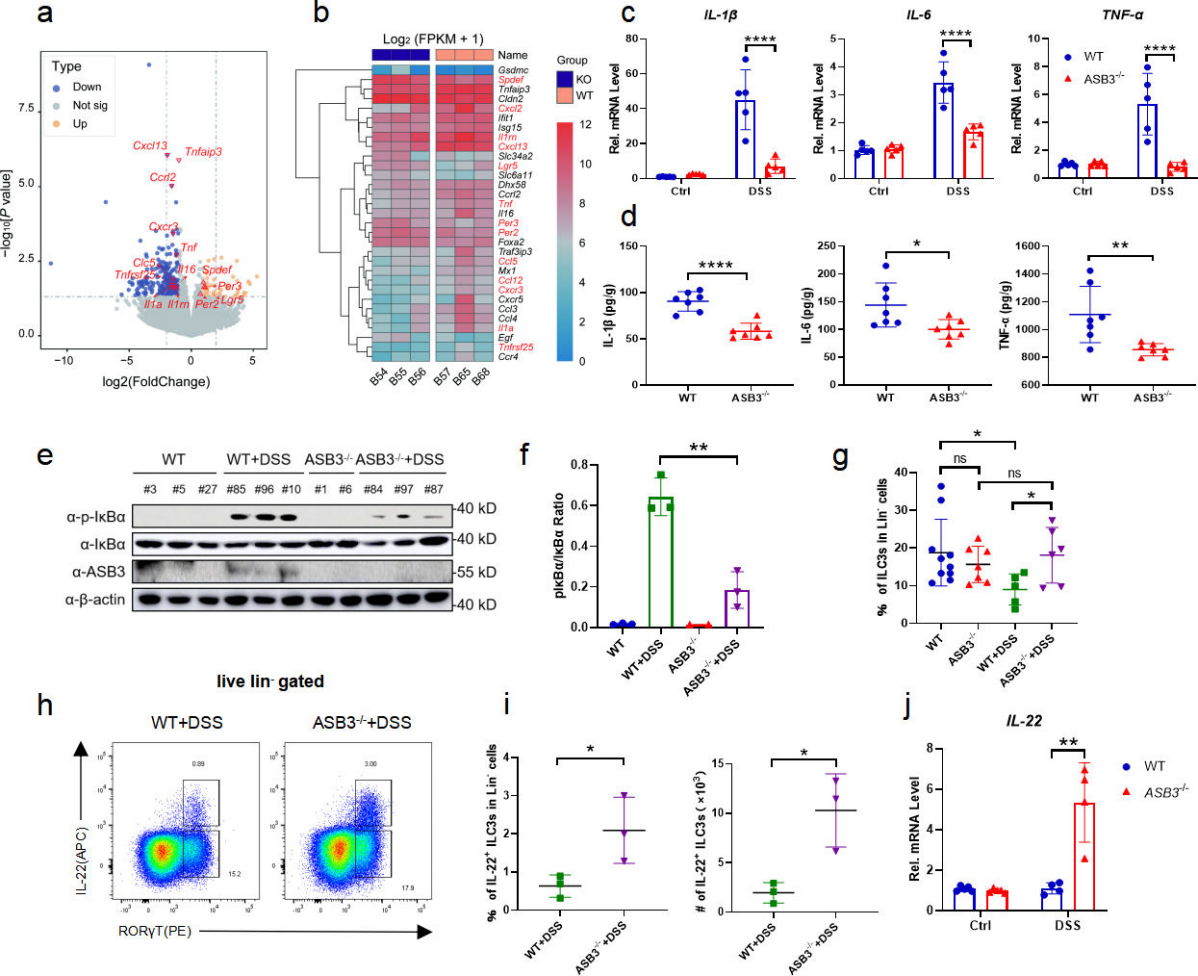
ASB3 deficiency inhibits the synthesis and release of proinflammatory cytokines. (**a and b**) Volcano plots and heatmap of RNA-seq data show upregulated or downregulated expression of genes in colon tissues from WT (*n* = 3) and ASB3^−/−^ mice (*n* = 3) treated with 3% DSS. (c) qPCR analysis of IL-1β, IL-6, and TNF-α mRNA expression in colon tissue sections from WT (*n* = 5) and ASB3^−/−^ mice (*n* = 5) treated with 3% DSS. (d) Colonic secretion of cytokines. (**e and f**) The expression of p-IκBα, IκBα, ASB3, and β-actin proteins in colon tissues from WT (*n* = 6) and ASB3^−/−^ mice (*n* = 5) treated with or without 3% DSS was detected by Western blotting. (g) Frequencies of ILC3s in cLPLs from WT and ASB3^−/−^ mice treated with or without 3% DSS. (h) Proportions of IL-22^+^ ILC3 cells in the colon of WT (ASB3^+/+^) and ASB3^−/−^ mice treated with 3% DSS. (i) Frequencies and absolute number of IL-22^+^ ILC3s in cLPLs from WT (*n* = 9) and ASB3^−/−^ (*n* = 9) mice treated with 3% DSS. (j) The mRNA expression levels of IL-22 were determined by qPCR assay. Differences were determined in panels b, c, d, and f by unpaired Student’s *t* test and in panels a and g by one-way ANOVA.

The development of IBD is closely related to intestinal cytokine storms (25). To determine whether ASB3 regulates the production of proinflammatory cytokines, we examined the production of IL-1β, IL-6, and TNF-α in the mouse colon (26). RNA-seq revealed that deletion of ASB3 resulted in lower levels of a large number of proinflammatory factors (Fig. 3a and b). As expected, the transcript levels and secretion levels of IL-1β, IL-6, and TNF-α in the colon of ASB3^−/−^ mice after the DSS challenge were much lower than those in WT mice (Fig. 3c and d). Moreover, ASB3 deficiency resulted in diminished phosphorylation of NF-κB-dependent proteins, exemplified by IκBα (Fig. 3e and f). These findings establish a link between ASB3 expression and increased IBD exacerbation and solidify that ASB3 expression may exacerbate colonic inflammation through excessive activation of immune signaling. To a great extent, mucosal immune cells directly alter the function of epithelial cells, thus adapting organ function to changing demands (27). The ILC population, which plays a critical regulatory role in intestinal inflammation, attracted our attention. We observed a significant decrease in the percentage of ILC3s in Lin− cells in colonic lamina propria lymphocytes (cLPLs) of WT mice but not in ASB3^−/−^ mice after administration of the DSS compared to controls (Fig. 3g). WT mice exhibited loss of the IL-22^+^ ILC3 subpopulation in colonic LPLs and reduced mRNA expression of IL-22 (Fig. 3h through j). ILC3-derived IL-22 is essential for tissue repair (28). Hence, we are also more convinced by this evidence that ASB3 expression is closely linked to exacerbated intestinal inflammation.

### ASB3 deficiency alters microbial composition during colitis

**Fig 4:**
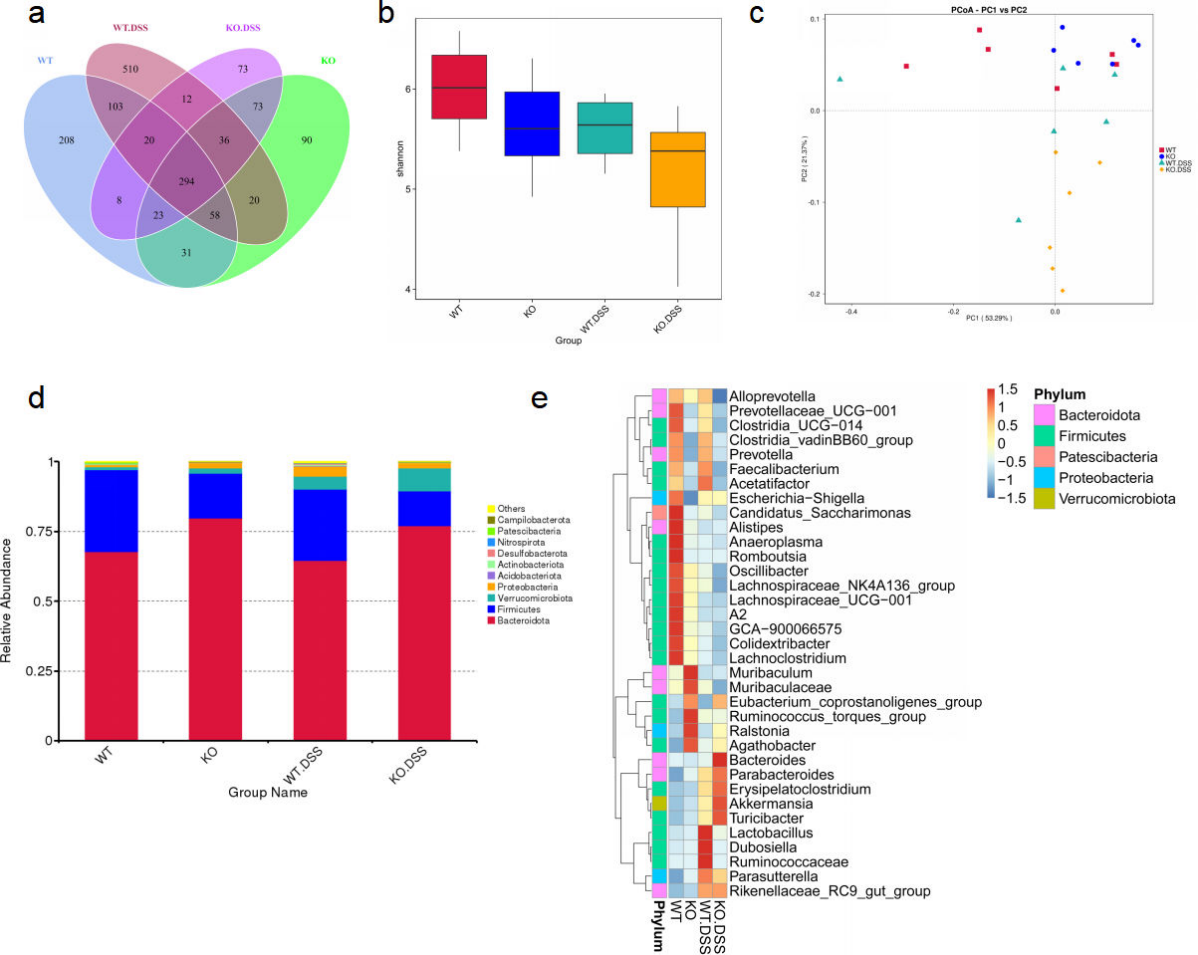
ASB3^−/−^ mice appear to have altered fecal microbiota. (a) Venn diagram depicting the number of microbiota at the bacterial taxa (phylum, class, order, and family) in the feces collected from WT and ASB3^−/−^ mice at day 0 or day 6 of DSS-induced colitis. (b) Analysis of the bacterial diversity of fecal microorganisms from DSS-induced colitis in WT and ASB3^−/−^ mice before and after. (c) Principal coordinate analysis (PCoA) of the weighted UniFrac distances of the fecal microbiota of WT and ASB3^−/−^ mice pre- and post-DSS challenge (*n* = 6 per group). (d) The relative abundance of microbial symbiont diversity was analyzed at the phylum level. (e) The relative abundance of microbial symbiont diversity was analyzed at the genus level.

Currently, the etiology of IBD is thought to be caused by inappropriate interactions between host cells and the gut microbiota secondary to increased inflammation involving the overgrowth of harmful opportunistic bacteria or pathogens and the loss of beneficial bacteria (29). To determine whether regulation of microbiota by ASB3 resulted in changes in specific bacteria, we performed a high-throughput sequencing analysis of 16S rDNA isolated from WT and ASB3^−/−^ mice before and after DSS challenge. These bacteria were originally derived from the same ASB3^+/-^ parents in our animal facility. The petal and box plots (Shannon index) show that the ASB3^−/−^ mice harbored microbiota with lower alpha diversity than WT mice (Fig. 4a and b). Notably, principal coordinate analysis revealed no segregation between genotypes at steady state. However, distinct colonies were formed in ASB3^−/−^ mice after DSS challenge (Fig. 4c). At the phylum level, compared with WT mice, the relative abundance of Bacteroidota and Verrucomicrobiota was elevated in ASB3^−/−^ mice before and after DSS treatment, and the relative abundance of Firmicutes was reduced, whereas the relative abundance of Proteobacteria was downregulated only after DSS treatment (Fig. 4d). Furthermore, at the genus level, the DSS-treated WT mice exhibited an increase in the relative abundance of *Lactobacillus*, *Dubosiella*, and *Ruminococcaceae,* and a decrease in the relative abundance of *Alistipes*, *Romboutsia*, *Lachnospiraceae*, and *Lachnoclostridium* compared with WT mice. In fact, most of the intestinal microbiota changes come primarily from members of Firmicutes. Of note, ASB3^−/−^ mice exhibited an increased relative abundance of *Bacteroides*, *Parabacteroides,* and *Akkermansia* (Fig. 4e). In summary, these data indicate that overactivation of ASB3 during the development of experimental colitis affects the composition of the intestinal microbiota and causes microbiota dysbiosis and that the latter may be due to the production of a large number of proinflammatory factors in these mice.

### Attenuated colitis in ASB3^−/−^ mice is partially dependent on altered microbiota

**Fig 5:**
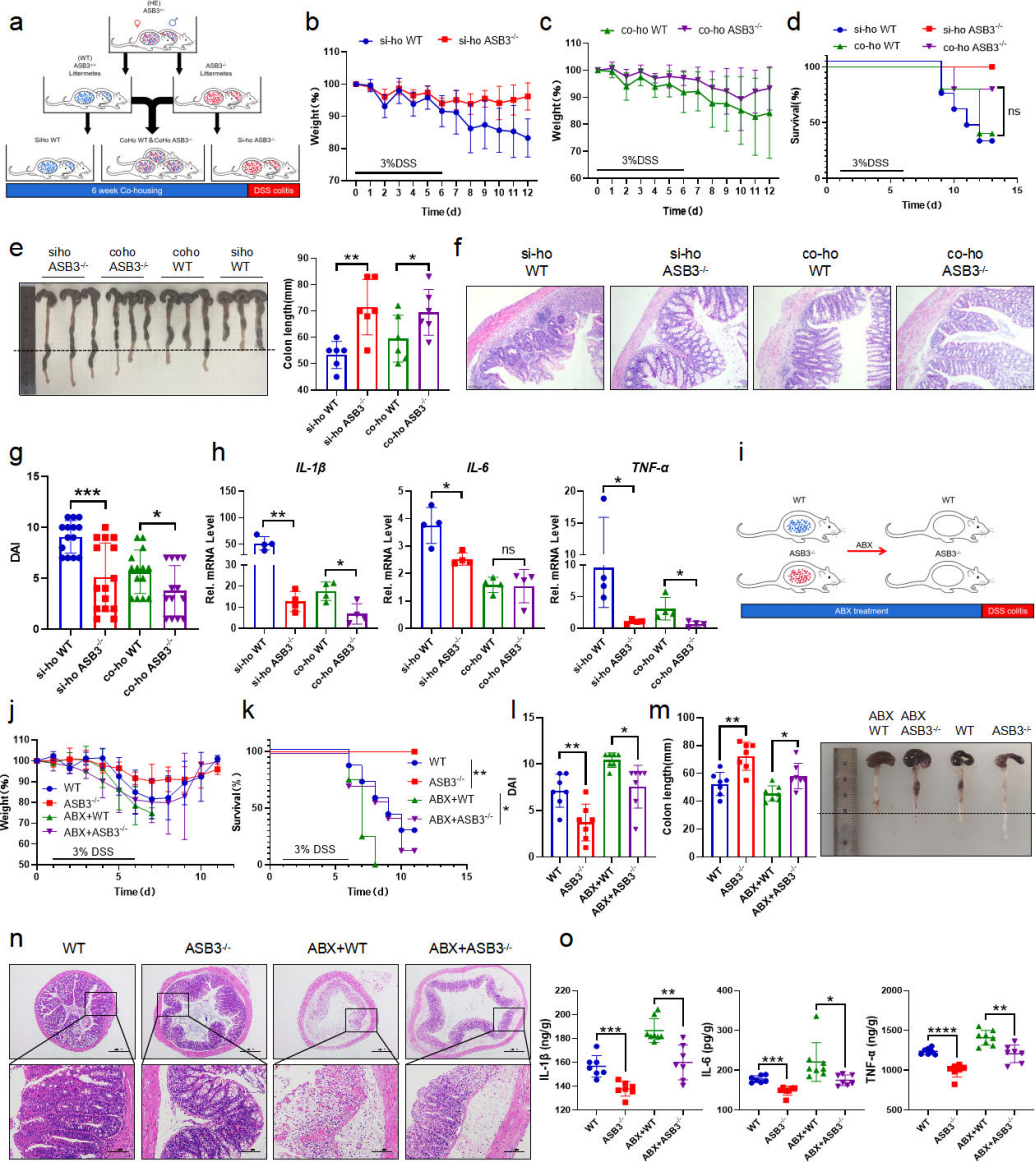
Commensal microbiota from ASB3^−/−^ mice enhances protection against DSS-induced experimental colitis in WT mice. (a) Schematic representation of the SiHo-CoHo strategy and DSS treatment. (**b–d**) Body weight (**b and c**) and survival (d) of SiHo-WT, SiHo-ASB3^−/−^, Coho-WT, or Coho-ASB3^−/−^ mice during DSS-induced colitis. (SiHo-WT, *n* = 7; SiHo-ASB3^−/−^, *n* = 5; Coho-WT, *n* = 6; and Coho-ASB3^−/−^, *n* = 5). (**e–g**) Colon length (e), representative H&E staining (f), and DAI (g) of WT, ASB3^−/−^, Coho-WT, or Coho-ASB3^−/−^ mice on day 8 after DSS induction. Scale bar, 50 µm. (h) The mRNA expression levels of IL-1β, IL-6, and TNF-α were determined by qPCR assay. (i) Schematic representation of the DSS challenge after ABX treatment. (j and k) Body weight changes (j) and survival (k) in WT and ASB3^−/−^ mice with or without combined ABX pretreatment for 5 days and 3% DSS for 6 days (WT, *n* = 7; ASB3^−/−^, *n* = 7; ABX + WT, *n* = 8; and ABX + ASB3^−/−^, *n* = 7). (**l–n**) DAI (l), colon length (m), and representative H&E staining (n) of WT, ASB3^−/−^, ABX + WT, or ABX + ASB3^−/−^ mice on day 8 after DSS induction. Scale bar, 50 or 100 µm. (o) Colonic secretion of cytokines. Differences were determined in panels e, g, h, i, m, and o by unpaired Student’s *t* test and in panels d and k by log-rank test.

To determine whether the slowing of colitis observed in ASB3^−/−^ mice was associated with the composition of the intestinal microbiota, we performed microbiota transfer studies by co-housing mice, which leads to the exchange of microbiota through coprophagia. Six- to eight-week-old WT and ASB3^−/−^ mice were either housed individually (SiHo mice) or cohoused (CoHo mice) for 6 weeks and then treated with 3% DSS (Fig. 5a). We found that SiHo WT mice and SiHo ASB3^−/−^ mice continued to have significantly different rates of weight loss after receiving DSS (Fig. 5b). However, ASB3^−/−^ mice cohoused with WT mice (CoHo ASB3^−/−^ mice) exhibited similar rates of weight loss as WT mice (CoHo WT mice) in the same littermates (Fig. 5c). Notably, CoHo ASB3^−/−^ mice showed acute phase death on day 9, a trend similar to that of WT mice after either littermates or non-littermates DSS administration (Fig. 5d). Furthermore, differences between CoHo ASB3^−/−^ and their WT cage companions were reduced in almost all measurements, including colon length, disease activity scores, and histopathological changes (Fig. 5e through g). We also demonstrated that microbial exchange somewhat alleviated IκBα phosphorylation and inflammatory factor (IL-1β, IL-6, and TNF-α) expression in the colons of CoHo WT mice (Fig. S1e) (Fig. 5h). We note that while the transferred microorganisms reduced the enteritis phenotype within the same litter of WT mice, the difference was narrowed but still existed. We speculate that the role in IBD pathogenesis of inappropriate host gene-microbe interactions may be causal. To test this, we first cleared the intestinal flora using a combination of antibiotics and then induced colitis using DSS (Fig. 5i). ABX-treated ASB3^−/−^ mice lost their resistance to DSS-induced colitis, experienced a considerable loss of body weight, and appeared to have died on day 6 (Fig. 5j and k). Interestingly, despite the microbiota depletion altering the colitis phenotype in ASB3^−/−^ mice, their active inflammatory manifestations, including DAI, colon length, and damage to colonic epithelial tissues remained lower than those observed in ABX-treated WT mice (Fig. 5l through n). In addition, ASB3 deficiency alleviated the secretion and production of inflammatory factors to varying degrees compared to control WT mice, whether the microbiota is depleted or not (Fig. 5o) (Fig. S1f). These data suggest that ASB3 expression may preferentially determine the substrate of intestinal inflammation, amplifying microbial signals through many inflammatory factors, thereby exacerbating intestinal barrier damage and the development of colonic inflammation.

### ASB3 specifically interacts with TRAF6

**Fig 6:**
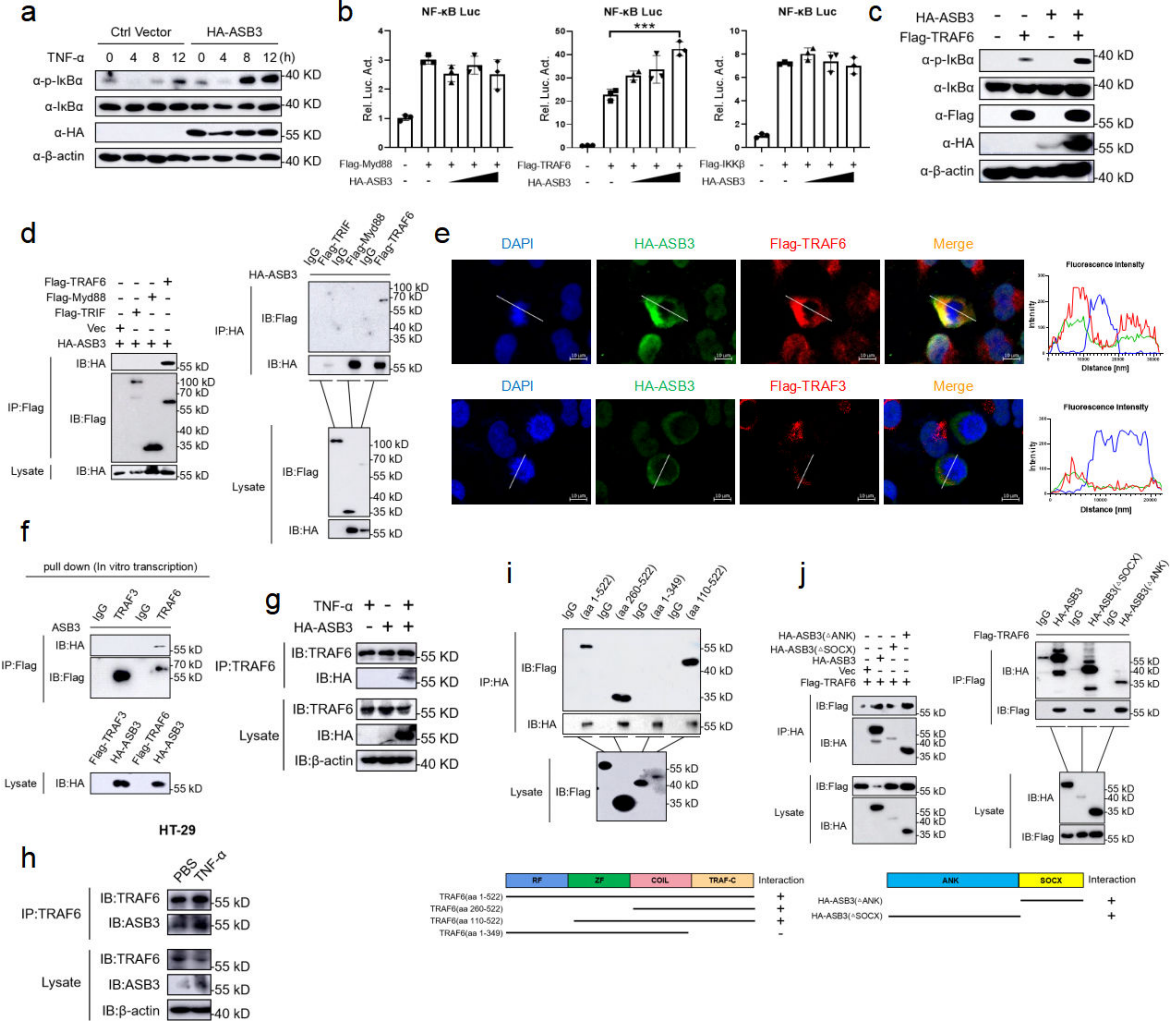
ASB3 specifically interacts with TRAF6. (a) HEK293T cells were transfected with HA-ASB3 plasmid or a vector control. Twenty-four hours after transfection, the cells were treated with TNF-α (100 ng/mL) for the indicated times. Cell lysates were separated by SDS-PAGE and analyzed by immunoblotting with the indicated antibodies. (b) HEK293T cells were transfected with the indicated plasmids along with control vector or increased amounts of ASB3 expression plasmids. Reporter assays were performed 24 h after transfection. (c) HEK293T cells were cotransfected with HA-ASB3 and Flag-TRAF6 plasmids or a vector control. (**d and f**) The indicated plasmids were transfected into HEK293T cells or transcribed *in vitro*. Then, coimmunoprecipitation (Co-IP) and immunoblotting analyses were performed with the indicated antibodies. (e) HEK293T cells were transfected with HA-ASB3, Flag-TRAF6, or Flag-TRAF3 plasmids for 24 h and then stained with Flag antibody or HA primary antibody and secondary antibody. The nuclei were stained with DAPI. The fluorescence intensity profile of DAPI (blue), HA-ASB3 (green), and Flag-TRAF6 or Flag-TRAF3 (red) was measured along the line drawn by ZEN Blue. Scale bars, 50 µm. (**g and h**) Semi-endogenous and endogenous Co-IP analysis of the interaction of ASB3 with TRAF6 in HEK293T or HT-29 cells treated with TNF-α for 24 h (100 ng/mL). (**i and j**) Co-IP analysis of the interaction of TRAF6 with ASB3 or its truncation mutants in HEK293T cells. Differences were determined in panel b by unpaired Student’s *t* test.

A recent study showed that overexpression of ASB17 increased the level of lipopolysaccharide (LPS)-mediated NF-κB activation in THP-1 cells (30). However, the mechanism of ASB3-induced NF-κB activation is unclear. To determine the role of ASB3 in NF-κB signaling pathway activation, we stimulated WT and ASB3-overexpressing 293T cells with TNF-α and found that overexpression of ASB3 significantly upregulated the phosphorylation level of IκBα (Fig. 6a). In Toll-like receptor or IL-1 signaling, Myd88-dependent NF-κB activation is linked by multiple signaling molecules, including TNF receptor-associated factor 6 (TRAF6), inhibitor kappa B kinase α (IKKα), inhibitor kappa B kinase β (IKKβ), TGF-β-activated kinase 1 (TAK1), and TAK1-binding protein 2 (TAB2) (31). We next used an NF-κB reporter gene to screen for key activation sites in inflammatory pathways. The results showed that ASB3 activated NF-κB reporter gene activity mediated only by TRAF6 but not by Myd88 and IKKβ (Fig. 6b). We also found that overexpression of ASB3 upregulated IκBα phosphorylation mediated by TRAF6 (Fig. 6c). TRAF6 is most widely described as a positive regulator of NF-κB signaling, and we speculate that ASB3 involvement in NF-κB activation may involve TRAF6 targeting. We then transfected labeled ASB3 and other signaling molecules into 293T cells and identified the proteins interacting with ASB3 by immunoprecipitation. TRAF6 protein, involved in the NF-κB signaling pathway, was identified as an interaction partner of ASB3 (Fig. 6d). Furthermore, confocal imaging confirmed the specific colocalization of ASB3 and TRAF6 (Fig. 6e). We also used *in vitro* transcription to further demonstrate that ASB3 interacts explicitly with TRAF6 but not TRAF3 (Fig. 6f). Semi-endogenous and endogenous IP experiments demonstrated an enhanced interaction between ASB3 and TRAF6 during TNF-α treatment (Fig. 6g and h). The same phenomenon was also confirmed in colonic tissue (Fig. S2a and b). Previous studies have shown that TRAF6 contains an RF domain, a COIL domain, a ZnF domain, and a TRAF-C domain (32). ASB3 is composed of two main functional structure domains, including the ANK domain and the SOCX domain. We found that ASB3 interacts only with the C-terminal structural domain of TRAF6 (Fig. 6i). Surprisingly, both the ANK domain and the SOCX domain were able to interact with TRAF6 (Fig. 6j). Taken together, these results demonstrate that TRAF6 is essential for ASB3-mediated NF-κB activation.

### ASB3 destabilizes TRAF6 by enhancing K48-linked polyubiquitination

**Fig 7:**
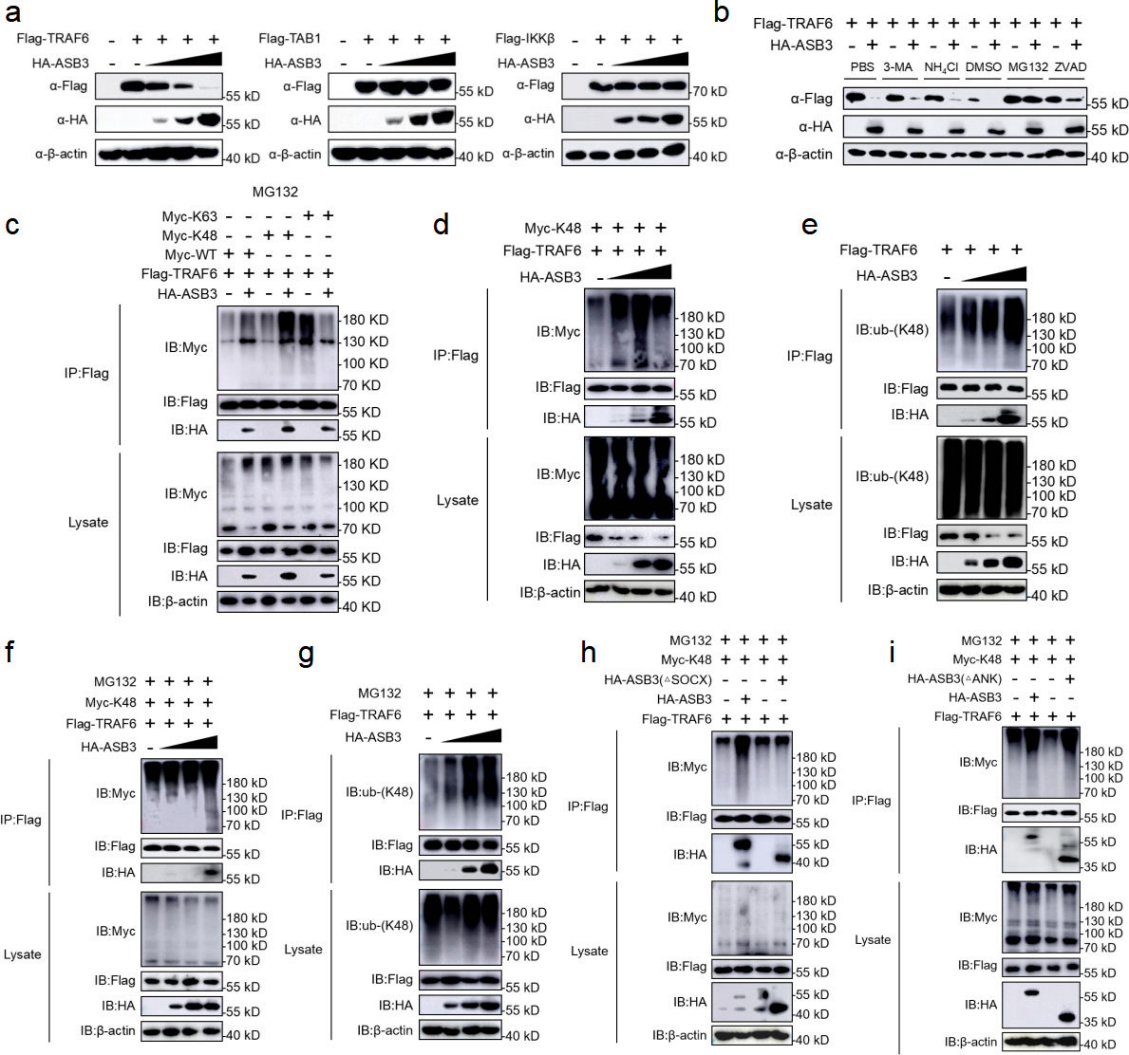
ASB3 promotes the K48-linked polyubiquitination of TRAF6. (a) HEK293T cells transfected with Flag-TRAF6, Flag-TAB1, or Flag-IKKβ with increasing amounts of HA-ASB3 for 24 h before immunoblotting analysis. (b) HEK293T cells were transfected with the indicated plasmids for 20 h and then treated with 3-MA (10 mM), NH_4_Cl (20 mM), MG132 (10 µM), or ZVAD (20 µM) for 6 h. The cell lysates were then analyzed by immunoblotting with the indicated antibodies. (c) HEK293T cells transfected with Flag-TRAF6, Myc-ubiquitin, or its mutants (KO, in which all but one lysine residues were simultaneously mutated to arginines [K-only]) together with control and ASB3 plasmids were pre-treated with MG132 (20 µM) for 6 h. The cells were then subjected to coimmunoprecipitation and immunoblotting analysis with the indicated antibodies. (d) HEK293T cells transfected with Flag-TRAF6 and Myc-K48 with increasing amounts of HA-ASB3 for 24 h. (e) HEK293T cells transfected with Flag-TRAF6 with increasing amounts of HA-ASB3 for 24 h. (f) HEK293T cells transfected with Flag-TRAF6, Myc-K48 together with control and ASB3 plasmids were pretreated with MG132 (20 µM) for 6 h, and the cells were then subjected to coimmunoprecipitation and immunoblotting analysis with the indicated antibodies. (g) HEK293T cells transfected with Flag-TRAF6 with increasing amounts of HA-ASB3 for 20 h and then treated with MG132 (20 µM) for 6 h. (**h and i**) HEK293T cells were transfected with the indicated truncated plasmids for 20 h and then treated with or without MG132 (20 µM) for 6 h, and samples were collected at the indicated times. The cells were used in ubiquitination assays with the indicated antibodies.

We previously observed that overexpression of ASB3 specifically upregulated TRAF6-mediated NF-κB reporter gene activity. We hypothesized that ASB3 might regulate the stability of TRAF6. To test this assumption, we cotransfected HA-tagged ASB3 with flag-tagged Myd88, TRAF6, or IKKβ and performed immunoblotting analysis. However, overexpression of ASB3 specifically downregulated TRAF6 expression but not TAB1 and IKKβ expression (Fig. 7a). The ubiquitin-proteasome and autophagy-lysosome pathways are the major systems controlling protein degradation in eukaryotic cells (19). We found that ASB3-mediated TRAF6 degradation was restored only by the proteasome inhibitor MG132 but not by the autophagy inhibitor 3-methylamide (3-MA), the lysosomal inhibitor ammonium chloride (NH_4_Cl), or the pan-Caspase inhibitor ZVAD (Fig. 7b).

To determine which ubiquitin chain of TRAF6 is modulated by ASB3, we analyzed ASB3-mediated ubiquitin mutants and the degradation of TRAF6. We found that ASB3 strongly mediates K48-linked polyubiquitination of TRAF6 (but not K63-) in a mammalian overexpression system and an IBD sample (Fig. 7c through e) (Fig. S2d). Adding MG132 led to the consistent conclusion that although TRAF6 protein expression was restored, the K48-linked polyubiquitin chains still accumulated (Fig. 7f and g). To further explore which structural domain of ASB3 is required for enhanced K48-linked polyubiquitination of TRAF6, we continued to use ASB3 truncates and examined their effect on TRAF6 ubiquitination. The results showed that removing the SOCX domain eliminated ASB3-mediated endogenous K48-linked polyubiquitination of TRAF6 (Fig. 7h). In contrast, truncation of the ANK domain does not eliminate TRAF6 exogenous K48-linked polyubiquitination (Fig. 7i). In conclusion, ASB3 enhances K48-linked polyubiquitination of TRAF6 through the interaction of its SOCX domain with the TRAF6 C-terminal domain.

### ASB3 disrupts TRAF6 stability in IECs and exacerbates lethal inflammation

**Fig 8:**
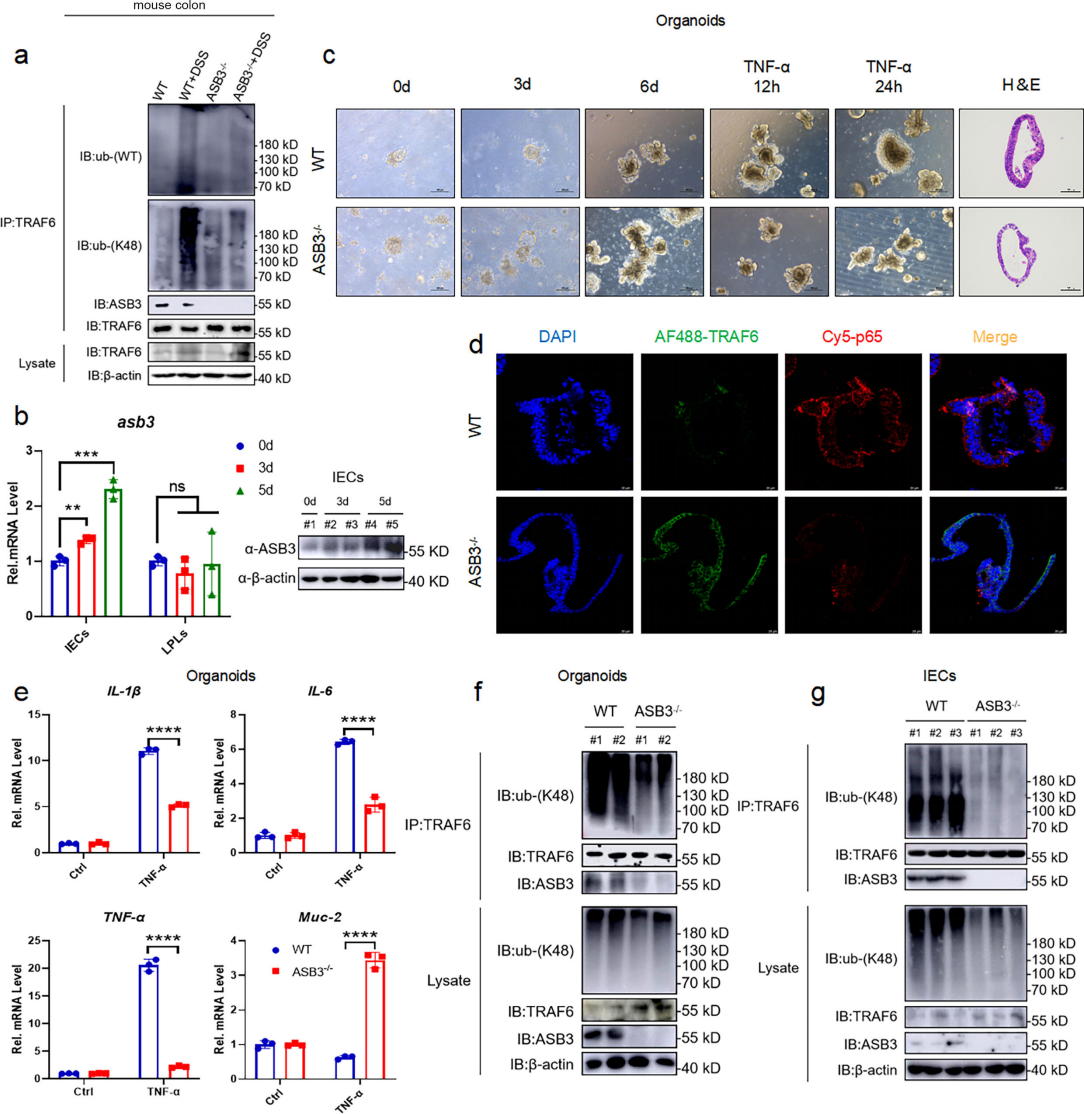
ASB3 promotes NF-κB signaling by limiting TRAF6 stability in IECs. (a) Total colonic tissue proteins from DSS-treated WT and ASB3^−/−^ mice. The samples were used in ubiquitination assays with the indicated antibodies. (b) Total RNA and protein of IECs and LPLs from dissociated colonic tissue from DSS-treated WT and ASB3^−/−^ mice. The samples were used in qPCR and immunoblotting assays. (c) Intestinal stem cells were harvested from untreated WT and ASB3^−/−^ mice, and the colon organoids were observed daily. Organoids were stimulated with mouse recombinant TNF-α (100 ng/mL) for 12 or 24 h on day 7. Scale bar, 100 µm. Representative H&E staining and IF in the colon organoid. Scale bar, 50 µm. (d) Colon organoids were analyzed on day 8 of culture and imaged by confocal microscopy after immunofluorescent staining for TRAF6 (green), NF-κB p65 (red), and DAPI (blue). Scale bar, 20 µm. (e) The mRNA expression of IL-1β, IL-6, TNF-α, and Muc-2 was measured in both TNF-α-treated colon organoids relative to controls. (f) Total colon organoid proteins from TNF-α-treated WT and ASB3^−/−^ mice. (g) Total protein of IECs from dissociated colonic tissue from DSS-treated WT and ASB3^−/−^ mice. The samples were used in ubiquitination assays with the indicated antibodies. Differences were determined in panel c by one-way ANOVA.

Since we observed that ASB3 deficiency eliminates K48-linked polyubiquitination of TRAF6 in mouse colon tissue (Fig. 8a), we hypothesize that the mechanism underlying the destabilizing regulation of TRAF6 by ASB3 may occur in IECs and trigger a previously reported TLR-Myd88/TRIF-independent NF-κB activation mediated by TRAF6^IEC-KO^ (33). To characterize the correlation between altered ASB3 in intestinal immune or non-immune cells and colitis, the IECs and LPLs from colon tissues of DSS-treated 3- and 5-day WT mice were isolated to perform qPCR and immunoblotting analysis. As expected, ASB3 expression was significantly upregulated in IECs after DSS treatment (but not LPLs) (Fig. 8b). Next, we constructed a model of colon organoid inflammation and found that after TNF-α treatment, organoids from ASB3^−/−^ mice exhibited less dark and disaggregated compared to those from WT mice (Fig. 8c). Next, we tested the effect of ASB3 deficiency on TNF-α-induced injury in colon organoids. Confocal analysis of treated and control organoids stained with TRAF6 and NF-κB p65 showed that administration of TNF-α to colon organoids exacerbated epithelial damage and downregulated TRAF6 expression, while ASB3 deficiency restored the expression of TRAF6 to stable levels (Fig. 8d). qPCR analysis also showed that deletion of ASB3 in colon organoids was effective in alleviating the overexpression of proinflammatory factors (Fig. 8e). To further confirm the effect of ASB3 on TRAF6 in IECs, we examined the types of ubiquitinated modifications. The results showed that ASB3 expression enhanced K48-linked polyubiquitination of TRAF6 in the colon organoids (Fig. 8f). In addition, similar results were obtained with IECs dissociated from mouse colon tissue (Fig. 8g). Collectively, these results reveal that the effect of ASB3 on intestinal phenotype mainly depends on its expression in epithelia and triggers massive release of proinflammatory factors by targeting TRAF6 destabilization.

## DISCUSSION

**Fig 9:**
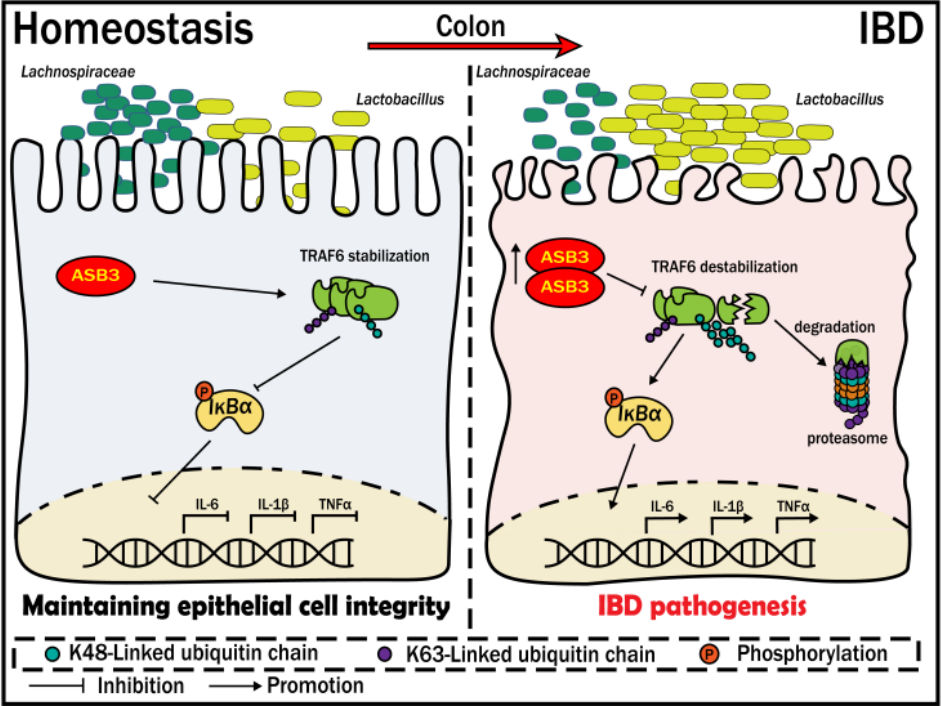
ASB3 aggravates intestinal epithelial inflammation and perturbs gut microbiota homeostasis by targeting TRAF6 destabilization. Under pathological conditions, ASB3 is upregulated in intestinal epithelial cells, leading to TRAF6 degradation via augmented K48-linked polyubiquitination. This results in the activation of TLR-independent NF-κB signaling, compromising the integrity of the intestinal epithelium and exacerbating the imbalance between the protective probiotic *Lachnospiraceae* and *Lactobacillus*. Our study underscores the significance of ASB3 as a crucial mediator for preserving intestinal mucosal homeostasis by interacting with TRAF6.

Ubiquitination plays an essential role in the pathogenesis and development of IBD. Ubiquitin-modifying enzymes (UMEs) coordinate the optimal ubiquitination of target proteins through synergistic effects, thereby maintaining gut homeostasis. However, in some reactions, these UMEs are aberrantly expressed and thus play a negative role. In the present study, we demonstrated that excessive activation of the E3 ligase ASB3 promotes the secretion of proinflammatory factors. In addition, we identified another key role of ASB3 in regulating gut microbiota imbalance by amplifying inflammatory signals. Mechanistically, ASB3 specifically enhanced K48-linked polyubiquitination, leading to TRAF6 destabilization. In this manner, proinflammatory factors are released in large quantities, the intestinal microecology and immune system are modulated, and damage due to inflammation is exacerbated. Thus, this study reveals a novel strategy for maintaining intestinal homeostasis by ASB3 protein-regulated inflammatory responses (Fig. 9).

A significant enhancement in ASB3 expression was reported in foci sites from UC and CD patients, compared to healthy sites (Fig. 1a through d and j). This phenomenon of exacerbated mucosal inflammation was confirmed in both our animal and organoid models (Fig. 1e through h). The role of ASB3 in intestinal diseases is poorly studied and is currently only found to be upregulated in COAD (Fig. S1a) (34). However, one study reported that ASB3 could inhibit CRC metastasis by delaying the epithelial-mesenchymal transition (35). Our current studies showed that ASB3^−/−^ mice had downregulated susceptibility to experimental colitis and preserved intestinal barrier function (Fig. 2). Interestingly, enhanced ASB3 expression is causally related to impaired intestinal homeostasis by the massive secretion of colonic proinflammatory factors. Thus, aberrant expression of ASB3 may have significant pathophysiological consequences.

ASB3 overexpression inhibited TRAF6 protein stability (Fig. 7a) but not transcription (Fig. S2c). We found that ASB3 specifically interacted with TRAF6 and degraded TRAF6 through K48-linked polyubiquitination (Fig. 7c through g). A previous study has reported that increased expression of zinc finger protein A20 inhibited TRAF6/NF-κB activation (36). TRAF6 autophagic degradation has also been shown to block NF-κB signaling (37). This may seem paradoxical at first; TRAF6 destabilization in our study still resulted in NF-κB expression and inflammatory factor secretion. Surprisingly, however, we noted in one experiment that mice lacking TRAF6 in IECs exhibited exacerbated DSS-induced inflammatory responses that TLR-related signaling molecules (Myd88 and TRIF) did not trigger (33). TRAF6 signaling independent of TLRs likely limits DSS-induced colitis not by mediating intrinsic functions involved in IEC homeostasis, but instead by inducing the release of epithelial cytokines such as TGF-β to exert direct or indirect anti-inflammatory functions (38). MALT1 has been reported to regulate STAT3 during IBD recovery to positively promote mucosal healing (39). This explains the upregulation of IL-22 expression in the colon, which may be achieved through interactions between IECs and ILCs. Numerous studies have shown that the TLR-induced NF-κB signaling pathway depends on the regulation of E3 ligases (40). A20 was shown to be an important regulator of NF-κB and have a bidirectional function in inflammatory stabilization (41). In fact, we found that ASB3-mediated destabilization of TRAF6 activated NF-κB *in vivo* and *in vitro*, which may be a novel mechanism for disrupting the homeostasis of inflammation maintained by TRAF6.

In the present study, we found a negative correlation between ASB3 expression and Lgr5 (Fig. 3a and b) (Fig. S2e). Recent reports have confirmed that the E3 ubiquitin ligase TRIM27 maintains intestinal homeostasis by activating Wnt signaling to promote the self-renewal of Lgr5^+^ ISCs (42). Intestinal homeostasis requires the integrity of the intestinal epithelial barrier to be maintained, and epithelial cells maintain dynamic homeostasis by coordinating with other intestinal cell populations and with cytokines (43). Whether ASB3 affects ISCs to drive epithelial cell proliferation and repair needs to be investigated in depth in the future with the help of organoid models. Immune cells in ASB3-deficient mice behaved normally at steady state, and intestinal ILC3 numbers were upregulated in inflammatory states (Fig. S3) (Fig. 3g). Our study also showed more severe intestinal inflammation accompanied by a reduced amount of IL22^+^ ILC3s, possibly limited by the degree of epithelial cell inflammation (Fig. 3i and j). The immune-related roles played by intestinal epithelial cells are primarily driven by gut-resident natural immune cells. These cell populations depend on the regulation of several molecules during IBD. DR3-driven loss of ILC3s in the large intestine is an essential factor contributing to the exacerbation of colitis (44), and mice lacking CD93 expression in ILC3s also exhibit impaired IL-22 production and increased colonic inflammation (45). In another study, metabolic reprogramming during intestinal LTi cell or ILC3 activation was dependent on the regulation of the immune checkpoint PD-1 (46). ASB3 has been reported to degrade TNF-R2 via the proteasome pathway (8), and whether there is a correlation between this and DR3 remains to be explored. Our data confirm that targeting ASB3/TRAF6 interactions largely affects the tissue repair function of ILC3s by disrupting the intestinal barrier. However, we currently have no direct evidence to suggest that ASB3 is involved in ILC3 regulation.

We observed that ASB3-deficient mice acquired colitis resistance by protecting more Bacteroidota while reducing the sudden increase in *Lactobacillus* (Fig. 4e). Intestinal immunity and homeostasis are regulated by epithelial cell/immune cell interactions and require fine-tuned communication between the host and microbes. The growth of certain pathogenic bacteria usually manifests as IBD-associated microbial dysbiosis at the cost of loss of protective commensal bacteria. Host genetic mutations often amplify this disorder. ANG1 deficiency antagonizes the growth of Lachnospiraceae by increasing the level of *α-Proteobacteria*, leading to dysbiosis of the intestinal ecology (47). Similarly, Nlrp12 deficiency led to a reduction in microbiota diversity, loss of protective intestinal commensal strains of *Lachnospiraceae*, and an increase in the highly pathogenic bacterium *Erysipelotrichaceae* (5). CARD9 deletion reduces members of *Lactobacillus reuteri*, *Allobaculum*, and the phylum Actinobacteria and weakens intestinal tryptophan catabolic function (48). Although the gut microbiota changes underlying IBD are not unique, most of the Bacteroidota identified in one study were depleted in IBD, and the abundance of *Streptococcus* and *Lactobacillus* was increased (49). Members of Muribaculaceae and Lachnospiraceae have been identified as significant utilizers of mucin monosaccharides, and members of Rikenomycetaceae and Bacteroidetaceae are also thought to have functions in the utilization of mucin monosaccharides (50). Interconversion of mucin monosaccharide-utilizing intestinal commensal bacteria caused by ASB3 deficiency before and after the DSS challenge also implies that ASB3 may have potential effects on the intestinal mucus layer.

### Conclusion

In this study, we report a previously unidentified function of ASB3 deficiency in ameliorating DSS-induced colitis. Our data suggest that high expression of ASB3 in intestinal epithelial cells under pathological conditions mediates TRAF6 destabilization, thereby inducing TLR-Myd88/TRIF-independent NF-κB activation and impaired intestinal barrier. ASB3 exacerbates the interaction between abnormal immune signaling and dysbiotic microbiota, creating a vicious cycle that leads to persistent inflammation and ecological dysregulation in the gut. This evidence increases our understanding of the pathogenesis of colitis. Interventions of ASB3-mediated proinflammatory pathway and ASB3-synergistic microbiota may have potential relevance for clinical IBD applications.

## Data Availability

The data sets analyzed during the current study are available from the corresponding author upon reasonable request. The 16S rRNA sequencing of the fecal microbiota has been deposited in the Sequence Read Archive with the BioProject accession number PRJNA1000707. The RNA sequencing of the colon tissues has been deposited in the Sequence Read Archive with the BioProject accession number PRJNA1058803.

## SUPPLEMENTAL MATERIAL

Supplemental figures mbio.02043-24-s0001.pdf Figures S1 to S3.

ASM does not own the copyrights to Supplemental Material that may be linked to, or accessed through, an article. The authors have granted ASM a non-exclusive, world-wide license to publish the Supplemental Material files. Please contact the corresponding author directly for reuse.
