# Supplementary material for: ASB3 expression aggravates inflammatory bowel disease by targeting TRAF6 protein stability and affecting the intestinal microbiota
Source: mBio. 2024 Aug 20;15(9):e02043-24. doi: 10.1128/mbio.02043-24 (PMC11389410; doi:10.1128/mbio.02043-24)
Supplement: Supplemental figures — Figures S1 to S3. [file mbio.02043-24-s0001.pdf]

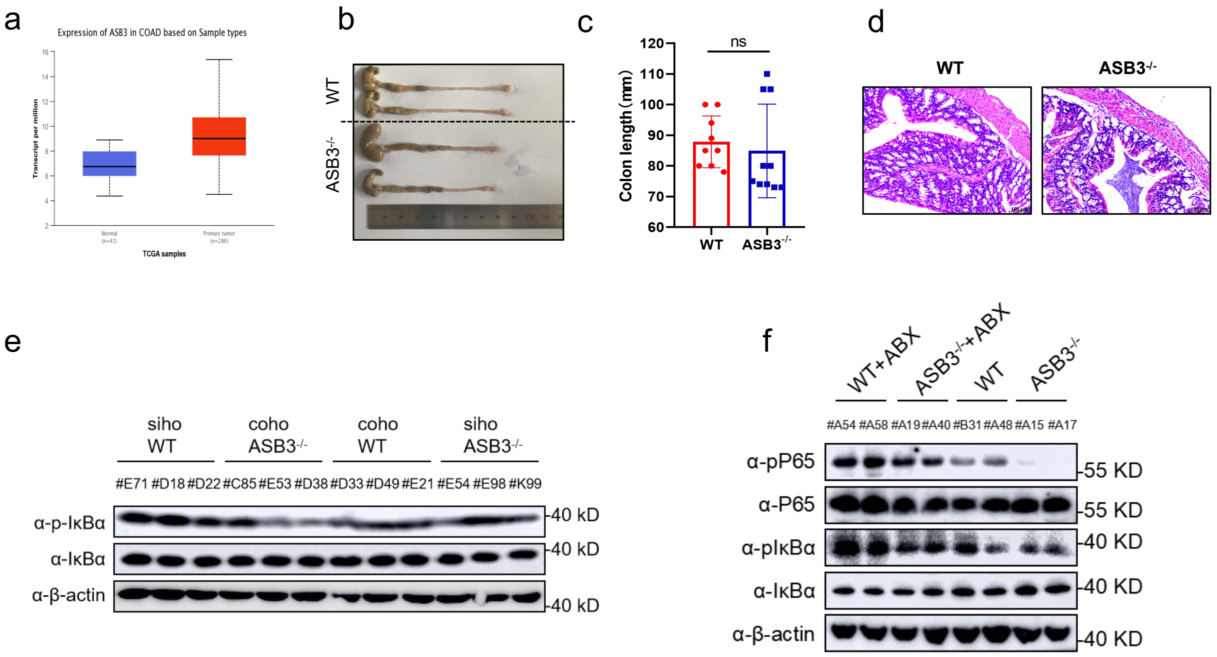

Supplementary Fig. 1 (**a**) The transcription levels of ASB3 in colon adenocarcinoma (TCGA). (**b, c**) Colon length of WT and ASB3$^{-/-}$ mice treated without DSS. (**d**) Representative images of pathological H&E stained colon sections collected. Scale bar, 100 μm. (**e, f**) The protein expression of p-IκBα, IκBα, and β-actin was detected by Western blotting.

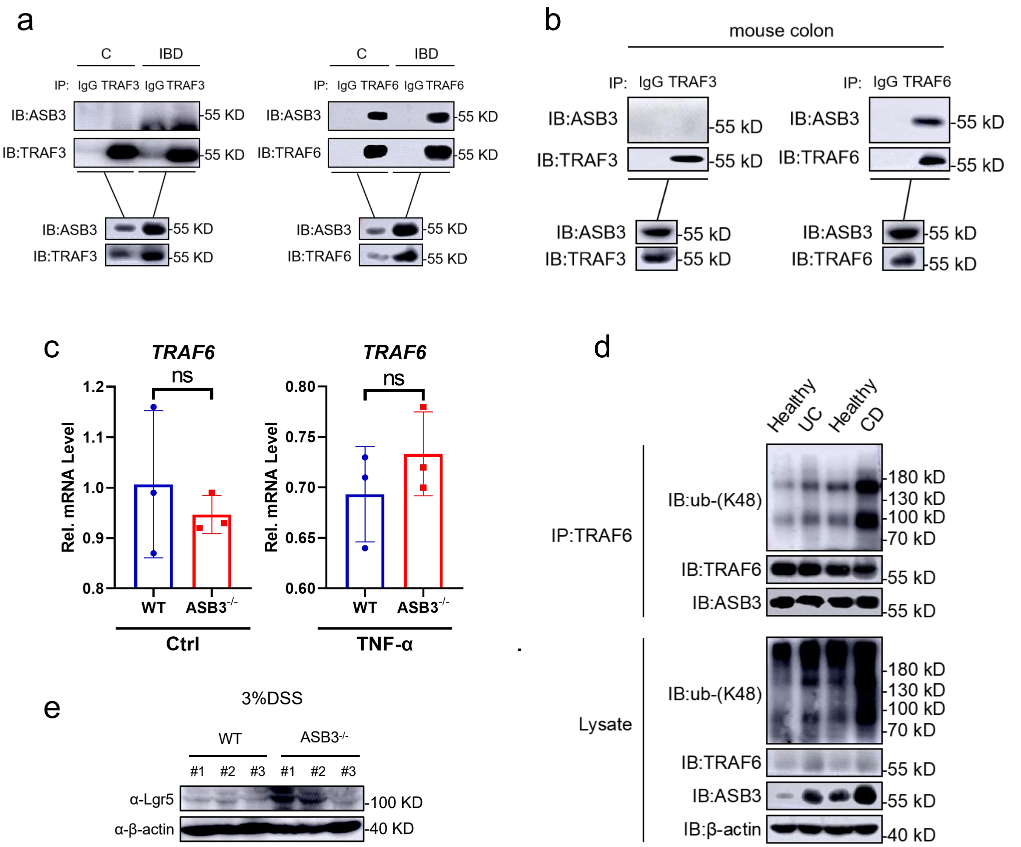

Supplementary Fig. 2 (**a**) Total colonic tissue proteins from healthy and IBD samples. (**b**) Total colonic tissue proteins from DSS-treated mice. The samples were used in Co-IP assays with the indicated antibodies. (**c**) The mRNA expression levels of TRAF6 in organoids were determined by qPCR assay. (**d**) Total colonic tissue proteins from healthy and IBD samples. The samples were used in ubiquitination assays with the indicated antibodies. (**e**) Western blotting was used to analyze Lgr5 protein expression in colons harvested from WT and ASB3$^{-/-}$ mice.

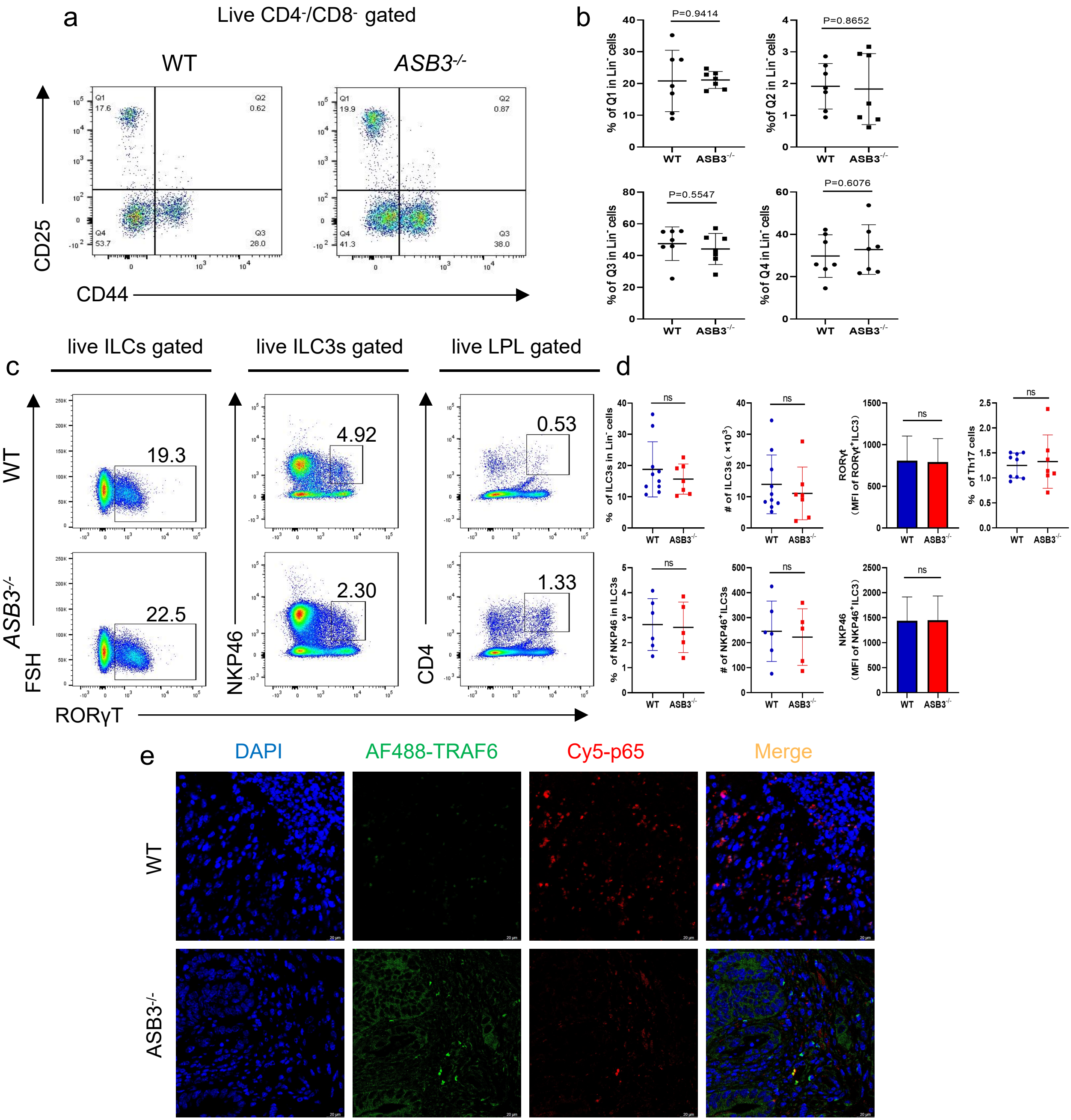

Supplementary Fig. 3 (**a, b**) Frequencies of CD44⁻CD25⁺, CD44⁺CD25⁺, CD44⁺CD25⁻ and CD44⁻CD25⁻ T-cells in thymuses from WT and ASB3$^{-/-}$ mice. (**c, d**) Frequencies, absolute number and MFI of RORγT⁺ ILC3s, NKP46⁺ ILC3s and Th17 cells in cLPLs from WT and ASB3$^{-/-}$ mice. (**e**) Colon tissues were analysed on day 8 from WT and ASB3$^{-/-}$ mice and imaged by confocal microscopy after immunofluorescent staining for TRAF6 (green), NF-κB p65 (red) and DAPI (blue). Scale bar, 20 μm.
